# Sex-Specific Effects of *AQP4* Gene Polymorphisms on Multiple Sclerosis Susceptibility and Response to Multidisciplinary Rehabilitation

**DOI:** 10.3390/ijms26188915

**Published:** 2025-09-12

**Authors:** Cristina Agliardi, Franca Rosa Guerini, Milena Zanzottera, Elisabetta Bolognesi, Domenico Caputo, Elisabetta Groppo, Marco Rovaris, Mario Clerici

**Affiliations:** 1 IRCCS Fondazione Don Carlo Gnocchi ONLUS, Via Capecelatro 66, 20148 Milan, Italy; cagliardi@dongnocchi.it (C.A.); mzanzottera@dongnocchi.it (M.Z.); ebolognesi@dongnocchi.it (E.B.); dcaputo@dongnocchi.it (D.C.); elisabetta.groppo@ospedaleniguarda.it (E.G.); mrovaris@dongnocchi.it (M.R.); mario.clerici@unimi.it (M.C.); 2 Clinical Neurology Unit, Department of Health Sciences, Ospedale San Paolo, ASST Santi Paolo e Carlo, University of Milan, 20142 Milan, Italy; 3 Pathophysiology and Transplantation Department, University of Milan, 20122 Milan, Italy

**Keywords:** multiple sclerosis (MS), relapsing–remitting, genetic, AQP4, polymorphism, SNP, rehabilitation, EDSS, outcome, gender

## Abstract

Multiple sclerosis (MS) is an inflammatory demyelinating disease of the central nervous system that affects young adults with different clinical phenotypes: relapsing–remitting MS (RR-MS), secondary progressive MS (SP-MS), and primary progressive MS (PP-MS). Aquaporin 4 (AQP4), a protein found in astrocytes, plays a crucial role in CNS functions. We investigated the possible association of three *AQP4* gene single-nucleotide polymorphisms (SNPs), rs2075575, rs162009, and rs335929, with MS risk and rehabilitation outcomes. SNPs were genotyped in 237 people with MS (pwMS), spanning all disease forms and enrolled in an intensive, multidisciplinary rehabilitation program, and 461 healthy controls (HCs). The *AQP4* rs2075575 GG genotype was significantly less frequent in male pwRR-MS compared to HCs (15.4% vs. 29.1%, *p* = 0.033, OR = 0.44), suggesting a protective role. Haplotype analysis identified the rs2075575(A)-rs162009(A)-rs335929(C) (A-A-C) haplotype as an MS risk factor, particularly in males (*p* = 0.001, OR = 2.70). Finally, the rs335929 SNP was significantly associated with EDSS improvement after rehabilitation (*p* = 0.011), with the CC genotype showing the highest mean ΔEDSS in pwRR-MS (*p* = 0.009), especially in males (*p* = 0.003). *AQP4* gene SNPs may influence both MS susceptibility and rehabilitation outcomes, with sex-specific effects. Further studies are needed to understand the mechanisms behind these associations and their potential for personalized treatment strategies in MS.

## 1. Introduction

Multiple sclerosis (MS), neuromyelitis optica (NMO), acute disseminated encephalomyelitis, transverse myelitis, and optic neuritis are inflammatory demyelinating diseases (IDDs) [1] of the central nervous system (CNS), primarily affecting young adults. The demyelination of nerve fibers gives rise to a wide range of neurological symptoms including cerebellar dysfunction, sensorimotor issues, visual disturbances, and gastrointestinal and genitourinary problems [2]. MS presents in various pathological forms. Relapsing–remitting MS (RR-MS) is characterized by episodes of neurological dysfunction, which may or may not lead to lasting disability. Up to 30% of RR-MS patients will experience a progression of disability, evolving into secondary progressive MS (SP-MS) within 10 to 20 years after the initial diagnosis. Finally, primary progressive MS (PP-MS), with a steady decline in neurological function from the onset of symptoms without distinct periods of relapse or remission, is diagnosed in approximately 15% of patients [3].

Aquaporin 4 (AQP4) is a water-selective channel protein, first described in 1994 [4]. It is highly expressed in astrocytes, particularly in the brain, spinal cord and optic nerve, where it is polarized at the end-feet of astrocytes, facing the vessel walls. This polarization of AQP4 is crucial for its function, and its loss is associated with a variety of brain pathologies [5]. AQP4 plays a vital role in several processes, including the regulation of extracellular volume, modulation of neuroexcitation, synaptic plasticity, neurogenesis, inflammatory responses, blood–brain barrier (BBB) permeability, and waste clearance within the glymphatic system [6]. In 2004, serum IgG antibodies targeting an epitope in the extracellular portion of AQP4 were identified in people with NMO [7], making AQP4 the first autoantigen linked to IDDs [8]. Anti-AQP4 IgGs are uncommon in people with MS (pwMS), but their presence is associated with a more severe disease progression and increased risk of optic neuritis [9]. Post-mortem analysis of MS brains showed that AQP4 expression is increased in more recent lesional foci, in particular at the periphery of the plaques [10]. AQP4 expression was found to be increased in a cuprizone-induced mouse model of MS, with a reduction in its polarization at astrocyte end-feet [11]. Magnetic resonance imaging (MRI) studies have shown increased water diffusion in both MS lesions and normal-appearing white matter, which is related to edema and an expanded extracellular space [12,13]. Notably, breakdown of the BBB or thickening of the basement membrane may contribute to ischemic-like conditions in MS, as supported by findings of ischemia-like lesions in pwMS [14].

The HLA-DRB1*15 allele has been repeatedly shown to be the major genetic risk factor for MS (with a *p*-value of approximately 10^−1900^) [15], but MS is believed to result from the combined contribution of many genetic factors [16]. In an attempt to shed further light on MS-associated genetic factors, we analyzed three *AQP4* gene single-nucleotide polymorphisms (SNPs) (rs2075575, rs162009, and rs335929) in pwMS with different forms of the disease who were undergoing inpatient multidisciplinary rehabilitation (IMR). Results were compared with matched healthy controls (HCs) to assess the potential involvement of these SNPs in both MS risk and rehabilitation outcomes.

## 2. Results

### 2.1. AQP4 SNP Genotypic, Allelic, and Haplotypes Distributions in Study Population

**Figure 1:**
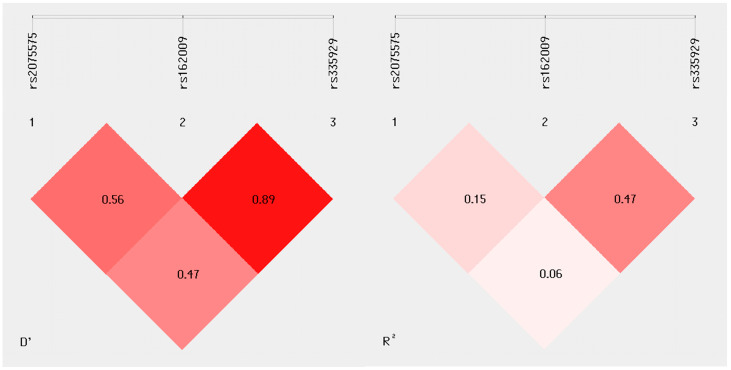
Linkage disequilibrium plots for *AQP4* gene polymorphisms rs2075575, rs162009, and rs335929. D′ and R^2^ values are reported for the study population.

All subjects included in the study were genotyped for the *AQP4* gene SNPs rs2075575, rs162009, and rs335929. The genotypic distribution of all SNPs was in Hardy–Weinberg equilibrium both in pwMS and in HCs. Linkage disequilibrium analysis results for the study population are reported in Figure 1.

The genotypic and allelic distributions of the three SNPs in pwMS and in HCs are reported in Table 1.

No significant differences were observed in the genotypic and allelic distributions of the three *AQP4* gene SNPs in the overall group of pwMS compared to HCs. Even when data were split by sex, no significative differences emerged.

PP-MS is clinically clearly distinct from SP-MS, which is the possible evolution of RR-MS; pwMS were thus split into two groups: PP-MS and RR-MS + SP-MS (RR-MS). The rs2075575 SNP GG genotype showed a protective effect in RR-MS males, being present in only 15.4% of patients compared to 29.1% of HCs (p_c_ = 0.033, OR = 0.44, 95% CI: 0.22–0.87) (Table 2).

Next, haplotype analyses were performed. The rs2075575 (A)-rs162009 (A)-rs335929 (C) (A-A-C) haplotype was identified as a risk factor for MS (considering the entire group), being present in 8.2% of pwMS compared to 5.3% of HCs (*p* = 0.03, Chi2 = 4.50, OR = 1.59, 95% CI: 1.03–2.47). When data were split by sex, the A-A-C haplotype remained a risk factor in males, as it was present in 12.1% of pwMS but in only 4.8% of HCs (*p* = 0.001, Chi2 = 10.07, OR = 2.70, 95% CI: 1.43–5.10). No significant differences in haplotype distribution were observed in females (Table 3).

Additionally, the rs2075575-rs162009-rs335929 haplotype did not influence the disease clinical phenotype (RR-MS (RR-MS + SP-MS) vs. PP-MS).

### 2.2. HLA-DRB1*15 Positivity

All individuals were genotyped for the HLA-DRB1*15 tag SNP rs3135388 to determine the presence of the MS-associated risk allele. This allele was detected in 62 of 237 pwMS (26.16%) and in 53 of 461 HCs (11.50%) (*p* = 1.5 × 10^−5^, OR = 2.7, 95% CI: 1.81–4.10), confirming that it was also a strong MS genetic risk factor in the study population.

### 2.3. AQP4 Gene SNPs and Their Impact on Multidisciplinary Rehabilitation Outcomes

Results of the Kolmogorov–Smirnov evaluation test showed than the expanded disability status scale (EDSS), the Modified Barthel Index (mBI), and the Numeric Rating Scale (NRS) scores were not normally distributed either at admission or at discharge.

The *AQP4* gene SNPs were assessed for their impact on multidisciplinary rehabilitation outcomes, measured by changes in EDSS, mBI, and NRS scores between admission and discharge. ΔEDSS, ΔmBI, and ΔNRS were calculated as follows: ΔEDSS = EDSS A − EDSS D; ΔmBI = mBI A − mBI D; and ΔNRS = NRS A − NRS D. A one-way ANOVA Kruskal–Wallis test revealed that rs335929 was associated with ΔEDSS (*p* = 0.026), particularly in the comparison between CC and AC genotypes (*p* = 0.021). This result was more pronounced in males (*p* = 0.013), especially in the comparison between CC and AC genotypes (*p* = 0.006).

Univariate linear regression analysis was then applied considering ΔEDSS as the dependent variable and the *AQP4* rs335929 SNP genotypes as the fixed factor and adjusting for EDSS A, age, disease duration, days of hospitalization, and HLA-DRB1*15 positivity. A significant association was found with *AQP4* rs335929 SNP (*p* = 0.011) in particular with a higher mean ΔEDSS for the rs335929 CC (−0.439) compared to the AA (−0.191) and the AC (−0.121) genotypes. The same analysis was applied with the splitting of pwMS according to sex. Results remained statistically significant in males (*p* = 0.011) but not in females. In particular, in males, a mean ΔEDSS of −0.561 was observed in rs335929 CC genotype carriers compared to a mean ΔEDSS of −0.054 for AC and −0.200 for AA genotype carriers. Pairwise comparisons for mean ΔEDSS in rs335929 genotypes showed statistically significant differences between subjects carrying CC vs. AA (*p* = 0.025) and CC vs. AC (*p* = 0.003) genotypes.

From a rehabilitative standpoint, individuals with RR-MS are clearly distinct from those with PP-MS and SP-MS [17]. Thus, to more finely evaluate possible correlations between *AQP4* SNPs and rehabilitation outcomes, pwMS were divided based on whether they had a diagnosis of RR-MS or of progressive (PP-MS/SP-MS) MS. A univariate linear regression analysis considering ΔEDSS as dependent variable and the *AQP4* rs335929 SNP as a fixed factor and adjusting for EDSS A, age, disease duration, days of hospitalization, and HLA-DRB1*15 positivity was applied. In the PP-MS/SP-MS group, ΔEDSS was observed to be influenced by the days of hospitalization (*p* = 0.025). In pwRR-MS, ΔEDSS was shown instead to be influenced by the *AQP4* rs335929 genotype (*p* = 0.009). In particular, higher ΔEDSS after rehabilitation was seen in CC carriers (−0.710) compared to both CA (−0.139) and AA (−0.243) carriers. Pairwise comparisons for mean ΔEDSS in rs335929 genotypes were statistically significant for CC vs. AA (*p* = 0.008) and CC vs. AC (*p* = 0.002).

The repeated measures analysis of variance for EDSS, mBI, and NRS scores was then applied, first considering the entire MS population. Results showed a general significant improvement for all the outcome indicators after multidisciplinary rehabilitation treatment (*p* < 0.001 for the three parameters). The same test was repeated, considering as the between-subject factor (grouping variable) the condition of “progressive” MS (PP-MS + SP-MS) or RR-MS. Results showed the presence of a correlation between EDSS outcome and the type of pathology, as a better outcome was seen in RR-MS patients compared to PP-MS/SP-MS patients (*p* = 0.043). The same test was applied again, considering as the between-subject factor the *AQP4* rs335929 genotype. Also in this case, the *AQP4* rs335929 genotype had a beneficial effect on EDSS outcome (*p* = 0.012).

**Figure 2:**
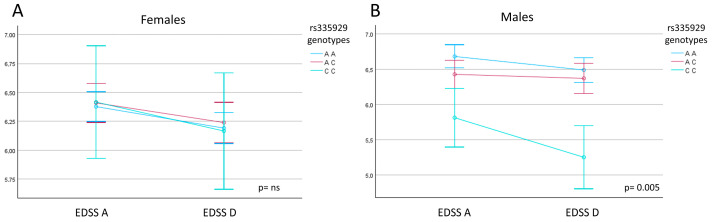
Repeated measures analysis of variance for EDSS scores. Mean EDSS at admission (EDSS A) and mean EDSS at discharge (EDSS D) are shown across different *AQP4* gene rs335929 genotypes. (**A**) Male pwMS, (**B**) female pwMS. *p* value is reported; error bars show standard deviation.

Results of the repeated measures analysis of variance performed with pwMS divided according to sex showed that the *AQP4* rs335929 genotype influences the EDSS outcome in male pwMS alone (*p* = 0.005) (female pwMS, *p* = 0.848) (Figure 2). Finally, repeated measures analysis of variance showed the mBI outcome to be influenced by the MS clinical phenotype (PP-MS/SP-MS vs. RR-MS) (*p* = 0.018) but not by any of the *AQP4* gene SNPs.

## 3. Discussion

In this study, we investigated the association between three single-nucleotide polymorphisms (SNPs) in the *AQP4* gene (rs2075575, rs162009, and rs335929) and both susceptibility to MS and response to multidisciplinary rehabilitation in pwMS. Our findings provide new insights into how *AQP4* genetic variability might influence disease risk and rehabilitation outcomes, with a particular focus on sex-specific effects.

Although no significant differences were observed in the overall genotypic and allelic distributions of the three SNPs between pwMS and HCs, stratified analyses revealed notable sex-related associations. Specifically, the rs2075575 GG genotype was significantly less frequent in male pwMS with a diagnosis of RR-MS, suggesting a potential protective role of this genotype in males. This observation is consistent with previous findings that highlighted a sex-dependent regulation of *AQP4* expression and function in the CNS, possibly influenced by hormonal factors [18,19]. Interestingly, a recent AI-based study suggested that AQP4 may be involved in the pathological accumulation of β-amyloid in Alzheimer’s disease, with a sex-dependent effect on early brain amyloid aggregation, potentially influenced by an AQP4 polymorphism-based risk score [20]. While our study focused on MS, these results are intriguing. Notably, the Rs2075575 A allele had been described as a risk factor both for NMO patients with anti-AQP4 antibodies [21] and for Parkinson disease in females [22]. Rs2075575 is located in the promoter region of the *AQP4* gene; variations in this region can potentially influence transcription factor binding, gene expression levels, and tissue-specific expression patterns. Supporting these results are data from a study on sudden infant death (SIDS) showing a reduction in AQP4-positive astrocyte density in the hippocampus in rs2075575 AG and AA carriers compared to GG carriers [23].

Haplotype analysis in our study identified the rs2075575 (A)-rs162009 (A)-rs335929 (C) (A-A-C) haplotype as a risk factor for MS in males alone, further supporting the hypothesis that sex-specific genetic backgrounds may contribute to MS susceptibility [24].

Beyond risk associations, our findings also indicate that *AQP4* polymorphisms, particularly the rs335929 SNP, influence the outcome of intensive multidisciplinary rehabilitation, as measured by changes in EDSS scores. Thus, the rs335929 CC genotype was associated with a significantly greater improvement in EDSS after rehabilitation, especially in male pwMS. This effect was confirmed by both univariate regression and repeated measures ANOVA and remained significant even after adjusting for potential confounders such as baseline EDSS, age, disease duration, hospitalization time, and positivity for the genetic risk allele HLA-DRB1*15. Importantly, the *AQP4* rs335929 CC genotype effect was most pronounced in pwRR-MS, while in progressive forms, rehabilitation outcomes were more influenced by hospitalization duration than genetic background. This apparent paradox, where the C allele of the *AQP4* rs335929 polymorphism within a rs2075575 (A)-rs162009 (A)-rs335929 (C) (A-A-C) haplotype is associated with an increased risk for MS yet correlates with better rehabilitation outcomes, may be explained by pleiotropic and context-dependent mechanisms. Thus, rs335929 location in the 3′ UTR suggests the ability of this SNP to influence gene expression by affecting microRNA binding sites or other regulatory elements, possibly modulating *AQP4* expression and AQP4 polarization. This would impact early astrocytic homeostasis, thereby increasing susceptibility to neuroinflammation and barrier dysfunction in the initial phases of the disease. However, once the disease is established, the same molecular configuration might enhance the neuroplasticity activity of AQP4 [25] or support reparative processes in response to environmental stimuli such as rehabilitation. These phenomena of genetic trade-offs are frequent in the evolutionary context [26].

Further supporting the role of AQP4 in MS pathogenesis and progression, a recent review suggests that the initial immune attack may target the ion and water homeostasis machinery in astrocytic endfeet, rather than myelin itself. This astrocyte dysfunction could then lead secondarily to myelin damage [27]. Moreover, animal studies have shown that voluntary physical exercise can accelerate glymphatic clearance, increase *AQP4* expression, and alter its distribution on astrocytes in aged mice [28]. Additionally, treadmill exercise has been found to influence the polar expression of *AQP4* in rats with local cerebral infarction [29]. These observations support our findings of a role for *AQP4* gene polymorphisms in both MS risk and rehabilitation outcomes, which could be mediated by the modulation of AQP4 expression and polarization, resulting in more efficient glymphatic clearance and astrocyte-mediated neurorepair mechanisms.

## 4. Materials and Methods

### 4.1. Study Population

Two hundred and thirty-seven pwMS (111 SP-MS, 92 RR-MS, and 34 PP-MS) diagnosed according to the revised McDonald diagnostic criteria [30] were enrolled in the study. All of the pwMS who were eligible for the present study were selected from a larger cohort from a previous study [31]. All pwMS underwent an inpatient multidisciplinary rehabilitation treatment at Neurorehabilitation Unit, MS Centre, IRCCS Fondazione Don C. Gnocchi (Milan, Italy). As already described in Groppo E. et al. [31], the admission criteria for the rehabilitation program included the presence of two or more moderate neurological disabilities upon clinical evaluation, along with functional deterioration within the previous six months. The intensive rehabilitation program included physiotherapy (motor rehabilitation) for all subjects, as well as occupational therapy for 78.3% of them. Additionally, 55.0% received speech and swallowing therapy, 22.9% underwent cognitive rehabilitation, 16.5% participated in respiratory therapy, 28.5% received formal psychological counseling, and 89.1% underwent pain management through physical therapy techniques such as massage, transcutaneous electrical nerve stimulation (TENS), electrical stimulation, and iontophoresis. Additional evaluations by specialists in cognitive, urological, ophthalmological, and respiratory medicine and other areas were conducted when necessary to define the rehabilitation plan that consisted of daily individual sessions of one or more activities from Monday to Saturday, totaling at least 500 min per week. The length of admission was determined after an intermediate multidisciplinary reassessment of the program and goals, involving physicians, therapists, and nurses, which took place after two to three weeks of admission. The mBI [32], EDSS [33], and NRS were used as rehabilitation outcomes and were assessed at both admission and discharge. Four hundred and sixty-one sex-matched HCs with no overt signs of neurological disease were also enrolled. HCs were intentionally selected with a higher mean age at enrollment than pwMS to ensure they were less likely to show any symptoms of the disease. The study was conducted according to the Declaration of Helsinki and was approved by the IRCCS Fondazione Don C. Gnocchi review board (protocol number #11_27/06/2019). All participants provided written informed consent. Clinical and demographic data of the study population are summarized in Table 4 (Table 4).

### 4.2. Sample Collection and DNA Extraction

Whole blood was collected in EDTA-containing Vacutainer tubes (Becton Dickinson Co., Rutherford, NJ, USA) and stored at −20 °C. Genomic DNA was extracted from peripheral blood mononuclear cells (PBMCs) using a standard phenol/chloroform method. DNA samples were stored at −20 °C until use.

### 4.3. AQP4 rs2075575, rs162009, and rs335929 SNP Description and Genotyping

The *AQP4* gene rs2075575, rs162009, and rs335929 SNPs were selected for analysis. Rs2075575 is located in intron 1, rs162009 lies within the promoter region, and rs335929 is located in the 3’ UTR region of the gene. Genotyping of these variants was conducted using allelic discrimination real-time PCR with pre-designed TaqMan assays (C__15863033_20 for rs2075575, C___1303573_30 for rs162009, and C___1303566_10 for rs335929) (Thermo Fisher Scientific, Waltham, MA, USA). The PCR protocol consisted of an initial hot start at 95 °C for 10 min, followed by 40 cycles of 15 s at 94 °C and 1 min at 60 °C. Fluorescence detection was performed at 60 °C. Reactions were carried out in 10 μL volumes using TaqMan Genotyping Master Mix (Thermo Fisher Scientific) on 96-well plates with a CFX96 instrument (Bio-Rad, Hercules, CA, USA). Control samples representing all possible genotypes, along with a negative control, were included in each run.

### 4.4. HLA-DRB1*15 Positivity

All pwMS were tested for HLA-DRB1*15 positivity, that is, the presence of at least one HLA-DRB1*15 allele, by genotyping the tag SNP rs3135388 [34] by allelic discrimination real-time PCR with the TaqMan C__27464665_30 assay (Thermo Fisher Scientific) and following the same procedure described above.

### 4.5. Statistical Analysis

Chi-squared or Fisher’s exact tests were employed to assess deviations in SNP genotype distribution from Hardy–Weinberg equilibrium. Chi-squared statistics were applied to 2 × N tables where appropriate to compare pwMS-HC and pwMS subtype differences in SNP genotype and allele distributions. Bonferroni correction was applied when required. As the calculated results for ΔEDSS = EDSS A − EDSS D, ΔmBI = mBI A − mBI D, ΔNRS = NRS A − NRS D were revealed to not be normally distributed after applying the Kolmogorov–Smirnov test, a one-way ANOVA Kruskal–Wallis test was applied to test the association of SNP genotypes with these quantitative clinical variables. Associations were evaluated using odds ratios (ORs) with 95% confidence intervals (CIs). Two-sided *p*-values were considered significant when <0.05; Bonferroni correction for multiple comparisons was applied when necessary (p_c_). Haplotype analyses and linkage disequilibrium graphs were generated using SHEsisPlus online software (http://shesisplus.bio-x.cn/SHEsis.html, accessed on 24 January 2025) [35]. Univariate linear regression analysis was applied to test the *AQP4* rs335929 SNP genotype’s influence on ΔEDSS considering EDSS A, age, disease duration, and days of hospitalization as covariates. Repeated measures analysis of variance for EDSS, mBI, and NRS was applied. For the between-subject factor (grouping variable), we first considered the condition of “progressive” MS (PP-MS + SP-MS) or RR-MS and then considered the *AQP4* rs335929 genotype, including as covariates EDSS, mBI, and NRS scores at admission, disease duration, sex, days of hospitalization, and HLA-DRB1*15 positivity. Statistical analyses were performed using SPSS software (v.29.0.1.0, IBM, Armonk, NY, USA), MedCalc (v. 11.5.0.0) and Jamovi (v. 2.6.44) (https://www.jamovi.org/cloud.html) (accessed on 20 January 2025).

## 5. Conclusions

The results herein suggest that *AQP4* polymorphisms play a role in MS pathogenesis and responsiveness to rehabilitation, potentially by modulating astrocytic water homeostasis, BBB integrity, neuroinflammatory processes, and neuroplasticity, processes critically involved both in MS progression and in recovery mechanisms [6]. The sex-specific effect observed may again be related to hormonal regulation of *AQP4* expression or differential immune responses between males and females.

While these findings are promising, some limitations must be acknowledged. First, the study population was limited in size, particularly after stratification by sex and MS subtype, which may affect the statistical power of subgroup analyses. Second, although the study focused on three SNPs, it is possible that other variants or regulatory elements of the *AQP4* gene may also contribute to MS risk and rehabilitation outcomes. Additionally, functional studies would be necessary to clarify how the identified SNPs or haplotypes affect *AQP4* expression or function in vivo. We understand that large-scale studies, including GWAS, may provide more comprehensive insights. However, candidate gene studies, provided they are based on solid scientific hypotheses and involve well-selected and homogeneous populations, can also be valuable. Future replication studies involving larger and more diverse cohorts, as well as longitudinal follow-up, will be crucial to confirm these results and to explore the utility of *AQP4* genotyping as a predictive tool for rehabilitation efficacy. Investigating the interplay between *AQP4* genotypes and imaging biomarkers (e.g., diffusion MRI or BBB integrity markers) could further elucidate the molecular mechanisms underlying the observed associations.

## Figures and Tables

**Table 1:** The *AQP4* rs2075575, rs162009, and rs335929 SNP genotypes and allele distributions in pwMS and HC.

rs2075575												
	Males						Females					
	**pwMS**		**HC**		***p* Value**	**OR (95% CI)**	**pwMS**		**HC**		***p* Value**	**OR (95% CI)**
**Genotype**	**N**	**(%)**	**N**	**(%)**			**N**	**(%)**	**N**	**(%)**		
**AA**	26	(27.4)	51	(26.0)	0.803	1.07 (0.61–1.86)	29	(20.4)	65	(24.5)	0.353	0.79 (0.48–1.29)
**GA**	52	(54.7)	88	(44.9)	0.118	1.48 (0.91–2.44)	73	(51.4)	138	(52.1)	0.898	0.97 (0.65–1.47)
**GG**	17	(17.9)	57	(29.1)	0.078	0.53 (0.28–0.97)	40	(28.2)	62	(23.4)	0.293	1.28 (0.80–2.04)
**Total**	95		196		0.106		142		265		0.467	
**Allele**												
**A**	104	(54.7)	190	(48.5)			131	(46.1)	268	(50.6)		
**G**	86	(45.3)	202	(51.5)			153	(53.9)	262	(49.4)		
**Total**	190		392		0.158	1.29 (0.91–1.82)	284		530		0.228	0.84 (0.63–1.12)
**rs162009**												
	**Males**						**Females**					
	**pwMS**		**HC**		***p* value**	**OR (95% CI)**	**pwMS**		**HC**		***p* value**	**OR (95% CI)**
**Genotype**	**N**	**(%)**	**N**	**(%)**			**N**	**(%)**	**N**	**(%)**		
**AA**	10	(10.5)	21	(10.7)	0.976	0.98 (0.43–2.16)	14	(9.9)	26	(9.8)	0.977	1.01 (0.50–1.98)
**GA**	45	(47.4)	90	(45.9)	0.817	1.06 (0.65–1.74)	64	(45.1)	113	(42.6)	0.639	1.10 (0.73–1.67)
**GG**	40	(42.1)	85	(43.4)	0.841	0.95 (0.58–1.56)	64	(45.1)	126	(47.5)	0.635	0.91 (0.60–1.34)
**Total**	95		196		0.973		142		265		0.884	
**Allele**												
**A**	65	(34.2)	132	(33.7)			92	(32.4)	165	(31.1)		
**G**	125	(65.8)	260	(66.3)			192	(67.6)	365	(68.9)		
**Total**	190		392		0.896	1.02 (0.71–1.48)	284		530		0.711	1.06 (0.78–1.44)
**rs335929**												
	**Males**						**Females**					
	**pwMS**		**HC**		***p* value**	**OR (95% CI)**	**pwMS**		**HC**		***p* value**	**OR (95% CI)**
**Genotype**	**N**	**(%)**	**N**	**(%)**			**N**	**(%)**	**N**	**(%)**		
**AA**	52	(54.7)	121	(61.7)	0.258	0.75 (0.46–1.24)	86	(60.6)	163	(61.5)	0.851	0.96 (0.63–1.46)
**CA**	35	(36.8)	66	(33.7)	0.595	1.15 (0.68–1.92)	50	(35.2)	92	(34.7)	0.919	1.02 (0.66–1.57)
**CC**	8	(8.4)	9	(4.6)	0.211	1.91 (0.68–5.24)	6	(4.2)	10	(3.8)	0.813	1.13 (0.37–3.17)
**Total**	95		196		0.314		142		265		0.967	
**Allele**												
**A**	139	(73.2)	308	(78.6)			222	(78.2)	418	(78.9)		
**C**	51	(26.8)	84	(21.4)			62	(21.8)	112	(21.1)		
**Total**	190		392		0.151	0.74 (0.50–1.12)	284		530		0.814	0.96 (0.68–1.37)

pwMS, people with multiple sclerosis; HC, healthy control; OR, odds ratio; CI, confidence interval; N, number.

**Table 2:** The *AQP4* rs2075575, rs162009, and rs335929 SNP genotype and allele distributions in the people with PP-MS, those with RR-MS (RR-MS + SP-MS), and HCs.

rs2075575												
Males							Females					
	**PP-MS**	**RR-MS (RR-MS + SP-MS)**	**HCs**	**PP-MS vs. RR-MS**	**PP-MS vs. HCs**	**RR-MS vs. HCs**	**PP-MS**	**RR-MS (RR-MS + SP-MS)**	**HCs**	**PP-MS vs. RR-MS**	**PP-MS vs. HCs**	**RR-MS vs. HCs**
**Genotype**	**N (%)**	**N (%)**	**N (%)**	***p* Value, OR (95% CI)**	***p* Value, OR (95% CI)**	***p* Value, OR (95% CI)**	**N (%)**	**N (%)**	**N (%)**	***p* Value, OR (95% CI)**	***p* Value, OR (95% CI)**	***p* Value, OR (95% CI)**
**AA**	5 (29.4)	21 (26.9)	51 (26.0)	0.823	0.746	0.872	3 (17.6)	26 (20.8)	65 (24.5)	0.805	0.556	0.422
**GA**	7 (41.2)	45 (57.7)	88 (44.9)	0.232	0.780	0.058	10 (58.8)	63 (50.4)	138 (52.1)	0.530	0.603	0.759
**GG**	5 (29.4)	12 (15.4)	57 (29.1)	0.292	0.954	0.033 *, 0.44 (0.22–0.87)	4 (23.5)	36 (28.8)	62 (23.4)	0.682	0.957	0.255
**Total**	17	78	196	0.323	0.943	**0.048**	17	125	265	0.808	0.798	0.463
**Allele**												
**A**	17 (50.0)	87 (55.8)	190 (48.5)				16 (47.1)	115 (46.0)	268 (50.6)			
**G**	17 (50.0)	69 (44.2)	202 (51.5)				18 (52.9)	135 (54.0)	262 (49.4)			
**Total**	34	156	392	0.546	0.865	0.125	34	250	530	0.907	0.697	0.235
**rs162009**												
**Males**							**Females**					
	**PP-MS**	**RR-MS (RR-MS + SP-MS)**	**HCs**	**PP-MS vs. RR-MS**	**PP-MS vs. HCs**	**RR-MS vs. HCs**	**PP-MS**	**RR-MS (RR-MS + SP-MS)**	**HCs**	**PP-MS vs. RR-MS**	**PP-MS vs. HCs**	**RR-MS vs. HCs**
**Genotype**	**N (%)**	**N (%)**	**N (%)**	***p* value, OR (95% CI)**	***p* value, OR (95% CI)**	***p* value, OR (95% CI)**	**N (%)**	**N (%)**	**N (%)**	***p* value, OR (95% CI)**	***p* value, OR (95% CI)**	***p* value, OR (95% CI)**
**AA**	2 (11.8)	8 (10.3)	21 (1.7)	0.822	0.844	0.932	1 (5.9)	13 (10.4)	26 (9.8)	0.630	0.672	0.846
**GA**	11 (64.7)	34 (43.6)	90 (45.9)	0.126	0.149	0.730	7 (41.2)	57 (45.6)	113 (42.6)	0.744	0.917	0.584
**GG**	4 (23.5)	36 (46.2)	85 (43.4)	0.093	0.117	0.804	9 (52.9)	55 (44.0)	126 (47.5)	0.500	0.675	0.515
**Total**	17	78	196	0.219	0.265	0.916	17	125	265	0.726	0.834	0.806
**Allele**												
**A**	15 (44.1)	50 (32.1)	132 (33.7)				9 (26.5)	83 (33.2)	165 (31.1)			
**G**	19 (55.9)	106 (67.9)	260 (66.3)				25 (73.5)	167 (66.8)	365 (68.9)			
**Total**	34	156	392	0.190	0.229	0.721	34	250	530	0.445	0.586	0.563
**rs335929**												
**Males**							**Females**					
	**PP-MS**	**RR-MS (RR-MS + SP-MS)**	**HCs**	**PP-MS vs. RR-MS**	**PP-MS vs. HCs**	**RR-MS vs. HCs**	**PP-MS**	**RR-MS (RR-MS + SP-MS)**	**HCs**	**PP-MS vs. RR-MS**	**PP-MS vs. HCs**	**RR-MS vs. HCs**
**Genotype**	**N (%)**	**N (%)**	**N (%)**	***p* value,** **OR (95% CI)**	***p* value,** **OR (95% CI)**	***p* value,** **OR (95% CI)**	**N (%)**	**N (%)**	**N (%)**	***p* value,** **OR (95% CI)**	***p* value,** **OR (95% CI)**	***p* value,** **OR (95% CI)**
**AA**	7 (41.2)	45 (57.7)	121 (61.7)	0.232	0.110	0.539	12 (70.6)	74 (59.2)	163 (61.5)	0.386	0.475	0.663
**AC**	8 (47.1)	27 (34.6)	66 (33.7)	0.352	0.283	0.878	5 (29.4)	45 (36.0)	92 (34.7)	0.618	0.681	0.803
**CC**	2 (11.8)	6 (7.7)	9 (4.6)	0.588	0.262	0.327	0 (0.0)	6 (4.8)	10 (3.8)	0.456	0.531	0.631
**Total**	17	78	196	0.459	0.177	0.564	17	125	265	0.517	0.611	0.847
**Allele**												
**A**	22 (64.7)	117 (75.0)	308 (78.6)				29 (85.3)	193 (77.2)	418 (78.9)			
**C**	12 (35.3)	39 (25.0)	84 (21.4)				5 (14.7)	57 (22.8)	112 (21.1)			
**Total**	34	156	392	0.233	0.078	0.368	34	250	530	0.293	0.386	0.596

PP-MS, primary progressive multiple sclerosis; RR-MS, relapsing–remitting multiple sclerosis; SP-MS, secondary progressive multiple sclerosis; HCs, healthy controls; OR, odds ratio; CI, confidence interval; N, number. * *p* value = *p* corrected for 2 degrees of freedom.

**Table 3:** Haplotype analysis of the AQP4 gene SNPs rs2075575, rs162009, and rs335929 in pwMS and HCs.

Haplotype	pwMS (M + F) (freq)	HCs (M + F) (freq)	Chi^2^	Pearson’s *p*	OR (95% CI)
**GGA**	118 (0.25)	222 (0.24)	0.113	0.74	1.05 (0.81–1.35)
**GAA**	51 (0.11)	106 (0.11)	0.170	0.68	0.93 (0.65–1.32)
**AGA**	192 (0.41)	389 (0.42)	0.365	0.55	0.93 (0.74–1.17)
**AAC**	39 (0.08)	49 (0.05)	4.498	0.03	1.60 (1.03–2.47)
**GAC**	67 (0.14)	133 (0.14)	0.021	0.88	0.98 (0.71–1.34)
**Global**			4.64	0.33	
**Haplotype**	**pwMS (F) (freq)**	**HCs (F) (freq)**	**Chi^2^**	**Pearson’s *p***	**OR (95% CI)**
**GGA**	74 (0.26)	126 (0.24)	0.52	0.47	1.13 (0.81–1.57)
**GAA**	34 (0.12)	58 (0.11)	0.20	0.66	1.11 (0.71–1.74)
**AGA**	114 (0.40)	228 (0.43)	0.63	0.43	0.89 (0.66–1.19)
**AAC**	16 (0.06)	30 (0.06)	2.5 × 10^−4^	0.99	1.00 (0.53–1.86)
**GAC**	42 (0.15)	71 (0.13)	0.30	0.58	1.12 (0.74–1.69)
**Global**			1.12	0.89	
**Haplotype**	**pwMS (M) (freq)**	**HCs (M) (freq)**	**Chi^2^**	**Pearson’s *p***	**OR (95% CI)**
**GGA**	44 (0.23)	92 (0.23)	0.006	0.93	0.98 (0.65–1.48)
**GAA**	17 (0.09)	48 (0.12)	1.40	0.24	0.70 (0.39–1.26)
**AGA**	78 (0.41)	165 (0.42)	0.06	0.81	0.96 (0.67–1.36)
**AAC**	23 (0.12)	19 (0.05)	10.07	0.001	2.70 (1.43–5.10)
**GAC**	25 (0.13)	62 (0.16)	0.71	0.40	0.81 (0.49–1.33)
**Global**			11.23	0.02	

pwMS, people with Multiple sclerosis; M, male; F, female; HCs, healthy controls; OR, odds ratio.

**Table 4:** Study population demographic and clinical data.

Population Characteristics			
	pwMS	HCs	*p*
**N**	237	461	
**Males/females**, (%/%)	95/142, (40.1/59.9)	196/265, (42.5/57.5)	0.54
**Age at enrollment** (years), mean ± SD	50.84 ± 12.13	69.95 ± 11.77	<0.001
**MS type**: RR-MS, PP-MS, SP-MS n (%)	92 (38.8), 34 (14.3), 111 (46.8)	n.a.	
**Age at onset** (years), mean ± SD	29.23 ± 11.42	n.a.	
**Disease duration** (years), median, IQR	20.0, 14.0	n.a.	
**Duration of admission**, (days) median, IQR	35.0, 13.0	n.a.	
**N of interventions**, mean ± SD	3.63 ± 1	n.a	
**EDSS I**, median, IQR	6.5, 1.5	n.a.	
**EDSS D**, median, IQR	6.5, 1.0	n.a.	
**BI I**, median, IQR	65.0, 27.25	n.a.	
**BI D**, median, IQR	75.0, 26.25	n.a.	
**VNS I**, median, IQR	5.0, 4.0	n.a.	
**VNS D**, median, IQR	3.0, 4.0	n.a.	

pwMS, people with multiple sclerosis; HCs, healthy controls; SD, standard deviation; RR-MS, relapsing–remitting multiple sclerosis; PP-MS, primary progressive multiple sclerosis; SP-MS, secondary progressive multiple sclerosis; IQR, interquartile range; EDSS I, expanded disability status scale; EDSS D, expanded disability status scale at discharge; BI I, Barthel index; BI D, Barthel index at discharge.

## Data Availability

The datasets used and/or analyzed during the current study are available from the corresponding author on reasonable request.

## Abbreviations

The following abbreviations are used in this manuscript:

MS	multiple sclerosis
CNS	central nervous system
RR	relapsing remitting
SP	secondary progressive
PP	primary progressive
BBB	blood–brain barrier
NMO	neuromyelitis optica
SNP	single-nucleotide polymorphism
EDSS	expanded disability status scale
EDSS	A EDSS at admission
EDSS	D EDSS at discharge
mBI	modified Barthel index
mBI A	mBI at admission
mBI D	mBI at discharge
NRS	Numeric Rating Scale
NRS A	NRS at admission
NRS D	NRS at discharge
IDD	inflammatory demyelinating disease
pwMS	people with MS
IMR	inpatient multidisciplinary rehabilitation
PCR	polymerase chain reaction
